# Identification of a Hypoxia-Related Gene Model for Predicting the Prognosis and Formulating the Treatment Strategies in Kidney Renal Clear Cell Carcinoma

**DOI:** 10.3389/fonc.2021.806264

**Published:** 2022-01-24

**Authors:** Xiang-hui Ning, Ning-yang Li, Yuan-yuan Qi, Song-chao Li, Zhan-kui Jia, Jin-jian Yang

**Affiliations:** ^1^ Department of Urology, the First Affiliated Hospital of Zhengzhou University, Zhengzhou, China; ^2^ Department of Nephrology, the First Affiliated Hospital, Zhengzhou University, Zhengzhou, China

**Keywords:** kidney cancer, hypoxia gene, prognosis, immunotherapy, nomogram

## Abstract

**Purpose:**

The present study aimed to establish a hypoxia related genes model to predict the prognosis of kidney clear cell carcinoma (KIRC) patients using data accessed from The Cancer Genome Atlas (TCGA) database and International Cancer Genome Consortium (ICGC) database.

**Methods:**

Patients’ data were downloaded from the TCGA and ICGC databases, and hypoxia related genes were accessed from the Molecular Signatures Database. The differentially expressed genes were evaluated and then the differential expressions hypoxia genes were screened. The TCGA cohort was randomly divided into a discovery TCGA cohort and a validation TCGA cohort. The discovery TCGA cohort was used for constructing the hypoxia genes risk model through Lasso regression, univariate and multivariate Cox regression analysis. Receiver operating characteristic (ROC) curves were used to assess the reliability and sensitivity of our model. Then, we established a nomogram to predict the probable one-, three-, and five-year overall survival rates. Lastly, the Tumor Immune Dysfunction and Exclusion (TIDE) score of patients was calculated.

**Results:**

We established a six hypoxia-related gene prognostic model of KIRC patients in the TCGA database and validated in the ICGC database. The patients with high riskscore present poorer prognosis than those with low riskscore in the three TCGA cohorts and ICGC cohort. ROC curves show our six-gene model with a robust predictive capability in these four cohorts. In addition, we constructed a nomogram for KIRC patients in the TCGA database. Finally, the high risk-group had a high TIDE score than the patients with low riskscore.

**Conclusions:**

We established a six hypoxia-related gene risk model for independent prediction of the prognosis of KIRC patients was established and constructed a robust nomogram. The different riskscores might be a biomarker for immunotherapy strategy.

## Introduction

Renal cell carcinoma is one of the most common malignant tumors in the urinary system (1). Clear cell renal cell carcinoma (ccRCC) is the most common type of RCC, and accounts for approximately 80% of cases, with a 10-year cancer specific-survival rate of 62% (2). Radical nephrectomy is the standard surgical treatment for localized and locally advanced ccRCC. However, there is a lack of sufficient effective treatment for metastatic ccRCC and its 5-year survival rate of 12% (1). Now the immune checkpoint inhibition agents have been recommended as the first line agents for metastatic RCC instead of targeted therapy (3). Constructing a prognostic model that can accurately predict the prognosis has become a key issue in the diagnosis and formulation of treatment strategies for renal cancer.

Nowadays, a few prognostic systems and nomograms have been developed based on the clinical characters (such as TNM stage and Fuhrman grade) and laboratory factors (such as hemoglobin, neutrophil count, and platelet count) (2). With the development of high-throughput sequencing technology, the nomogram based on the RNA-seq data or methylation data have been constructed (4–9). These nomograms have not only improved the capability of predicting the prognosis of ccRCC but have also indicated the pathogenic mechanism of ccRCC which may help to develop drug candidates.

VHL gene mutation plays a central role in the initiation and progression of ccRCC (10, 11). As VHL gene is a key regulator of hypoxia, and hypoxia related genes can facilitate tumorigenesis (12), whether the hypoxia pathway could predict the prognosis of ccRCC was interesting. Now, we used the TCGA and ICGC data to investigate the role of hypoxia pathway in predicting the prognosis of ccRCC, and our results constructed a hypoxia related genes model and a nomogram to improve the prognostic value of ccRCC based on bioinformatics approaches. We also investigate the role of our prognosis model in the immunotherapy strategy.

## Materials and Methods

### Patients and Public Datasets

RNA-seq data of 533 KIRC and 78 adjacent normal kidney tissues were downloaded from The Cancer Genome Atlas (TCGA) database (https://portal.gdc.cancer.gov/) in October 2018. These original data were processed according to the guidelines and the data were merged into a single expression file for further study. All these expression data were used for screening the differential expression genes between normal and KIRC tissues. The clinical data of KIRC patients were also obtained, and 512 patients who had identified information of age, gender, clinical stage, TNM stage, overall survival status, and survival time were finally enrolled in the prognosis study (**Supplementary File**; **Supplementary Table**). The International Cancer Genome Consortium (ICGC) cohort (RECA-EU project, 91 patients) clinical information and RNA expression data were downloaded from the ICGC data portal in April 2020 (**Supplementary File**, **Supplementary Table**). The TCGA was used as a public open database, and the relevant information retrieved from it did not require further ethical approval.

### Screening of Differential Expression Hypoxia Genes (DEHGs)

RNA-seq data were standardized using the “limma” package of R software. Then differentially expressed genes (DEGs) were evaluated using the “DESeq” package. Genes with adjusted p-value <0.05 and absolute log2 fold change (FC) >2 in normal and tumors tissues were considered as DEGs. The hypoxia related genes were downloaded from the Molecular Signatures Database (https://www.gsea-msigdb.org/gsea/msigdb/cards/HAL LMARK_HYPOXIA.html). The hypoxia related genes in DEGs and the ICGC genes list were considered as differential expression hypoxia genes (DEHGs).

### Prognosis Related Genes Filtering and Gene Risk Model Construction

The whole 512 patients were named Total TCGA cohort. Total TCGA cohort was then divided into a discovery TCGA cohort (256 patients) and a validation TCGA cohort (256 patients) by a ratio of 1 to 1 randomly (**Supplementary File**). The discovery TCGA cohort was used for constructing the hypoxia genes risk model. Firstly, univariate Cox expression was used to assess the prognosis role of these DEHGs. After identifying the prognosis related DEHGs, LASSO (least absolute shrinkage and selection operator) regression was used to select a panel of genes, and the analysis was performed using “glmnet” package in R software. Lastly, the genes which were extracted by lasso regression, were enrolled in multivariate Cox regression to further screen the prognosis related DEHGs and then construct a risk model. Riskscore was calculated according to the coefficient and expression value of each DEHG which had the significant meaning in multivariate Cox regression.

### Genes Risk Model Validation, Nomogram Construction and TIDE Score Calculation

Risk-groups were deemed as two categorical variables (high risk-group and low risk-group) according to the median value of riskscore. Kaplan–Meier curve and log-rank test were used to assess the prognosis role of risk-group. Next, univariate and multivariate Cox regression were used to identify whether riskscore was an independent factor for the prognosis of KIRC patients. In the Cox regression analysis, gender, T stage, N stage, M stage, clinical stage, and risk-group were considered as categorical variables. Meanwhile, age and riskscore were considered as continuous variables. Validation TCGA cohort, total TCGA cohort, and ICGC cohort underwent the same analysis procedure to validate the genes risk model. Moreover, Receiver operating characteristic curve (ROC) analysis was performed to assess the predictive accuracy of the riskscore in all these four cohorts. In addition, a nomogram was established based on the total TCGA cohort for clinical application. Statistical analyses were performed using R software. Tumor Immune Dysfunction and Exclusion (TIDE) score was calculated online (HTTP://tide.dfci.harvard.edu/) (13). All tests were two-tailed, and a P-value of <0.05 was considered statistically significant.

## Results

### Data Processing and Screening of Differentially Expressed Genes

A total of 5,825 DEGs were screened (3,370 upregulated and 2,455 downregulated) from a total of 34,827 genes using the adjust p-value <0.05 and log FC (fold change) >1 threshold (**Supplementary File**). Two hundred hypoxia-related genes were acquired from the Molecular Signatures Database. Finally, 72 genes DEHGs were identified both in total TCGA and ICGC cohorts (**Supplementary File**; **Supplementary Figure**).

### Screening of Prognostic DEHGs

The processed survival data of each tumor sample in the training set were subjected to univariate Cox proportional hazards regression analysis, in which the significant threshold was set at value <0.05. Therefore, 15 prognosis-related DEHGs containing 10 risky genes and 5 protective genes were identified (**Supplementary File**).

### Establishment and Validation of the 6-DEHGs Prognosis Model

**Figure 1:**
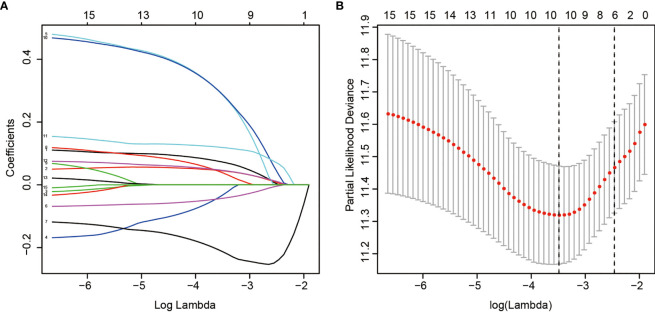
LASSO regression analysis of 15 DEHGs. Ten-fold cross-validation was applied to calculate the best lambda, which leads to a minimum mean cross-validated error **(A)**. A total of 6 DEHGs were adopted for the LASSO model **(B)**.

**Figure 2:**
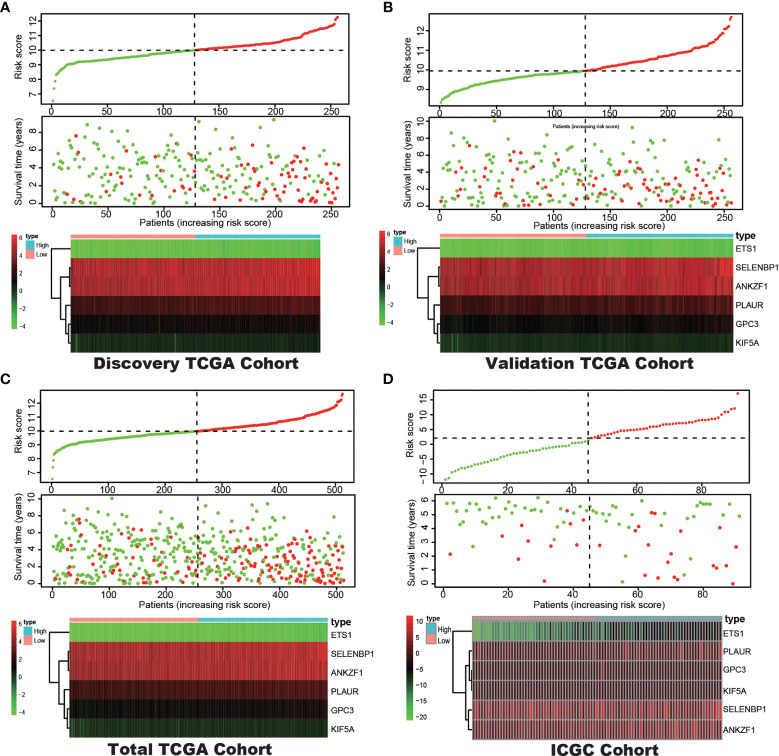
Correlation between the prognostic model and the overall survival (OS) of patients in the Discovery TCGA Cohort **(A)**, the Validation TCGA Cohort **(B)**, the Total TCGA Cohort **(C)** and the ICGC Cohort **(D)**. The distribution of riskscore (upper), survival time (middle) and hypoxia genes expression levels (below). Patients were classified into low-risk and high-risk groups by using the median score as a cut-off value. The red dots and lines represent the patients in high-risk groups. The green dots and lines represent the patients in low-risk groups.

**Figure 3:**
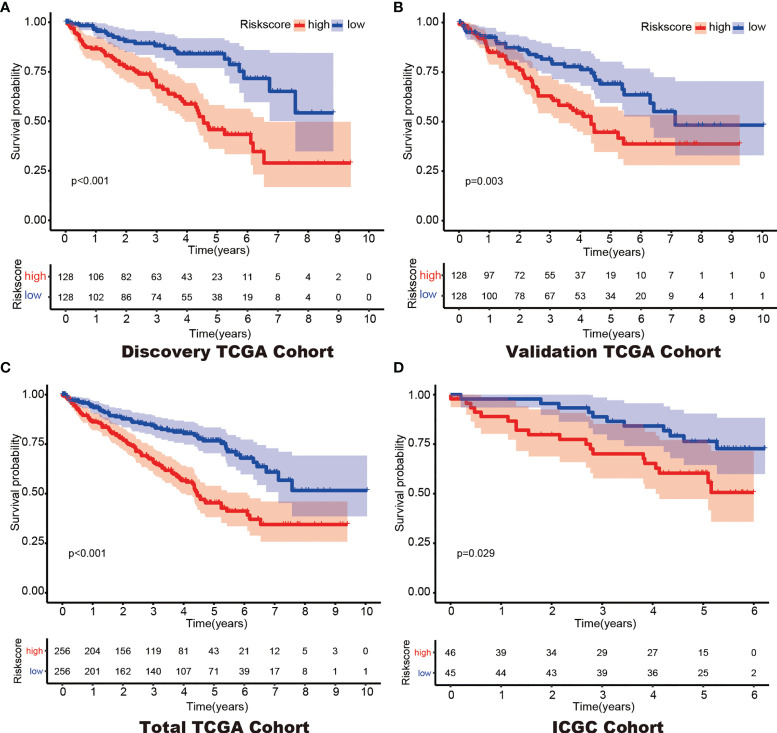
Kaplan–Meier survival of the 6-gene prognostic model in the Discovery TCGA Cohort **(A)**, the Validation TCGA Cohort **(B)**, the Total TCGA Cohort **(C)** and the ICGC Cohort **(D)**.

LASSO regression with tenfold cross validation was performed to further screen the DEHGs that significantly correlated with the prognosis of ccRCC patients. The optimal lambda value was obtained from the minimum partial likelihood deviance (**Figures 1A, B** **)**. Then, the optimal 6-DEHG model was obtained which contained *GPC3*, *KIF5A*, *PLAUR*, *ANKZF1*, *ETS1*, and *SELENBP1*. The Cox coefficients of the DEHGs were obtained from the multivariate Cox proportional hazards regression analysis. Then, the riskscore was constructed based on the coefficients and categorical values of expression level as the following: Riskscore = (0.1611 ∗ GPC3) + (0.0781 ∗ KIF5A) + (0.2598 ∗ PLAUR) + (0.4296 ∗ ANKZF1) − (0.2765 ∗ ETS1) + (0.4578 ∗ SELENBP1). The riskscore of each tumor patient in the discovery TCGA cohort was calculated and the patients were divided into a high risk-group and a low risk-group based on the median riskscore (**Figure 2**). Then, the other three cohorts were also divided into a high risk-group and a low risk-group based on the median riskscore of each cohort individually. Patients with the high risk-group presented with poorer survival (**Figure 3A**) and it had been validated in the other three cohorts (**Figures 3B–D**).

### Construction of a 6-DEHG Prognosis Model-Based Nomogram

**Figure 4:**
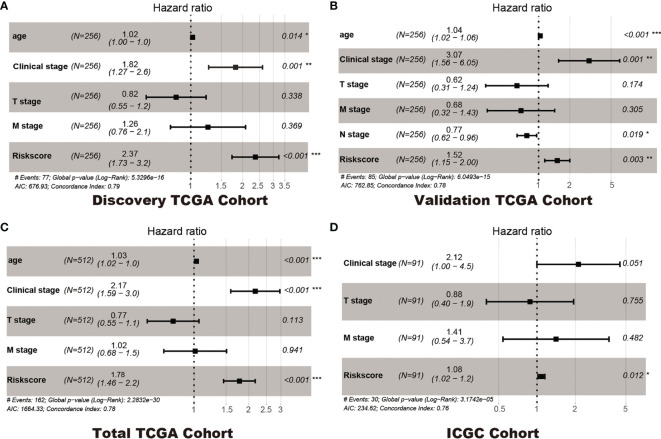
Multivariate Cox regression analyses. Discovery TCGA Cohort **(A)**, Validation TCGA Cohort **(B)**, Total TCGA Cohort **(C)** and ICGC Cohort **(D)**. *p < 0.05, ** p < 0.01, *** p < 0.001.

**Figure 5:**
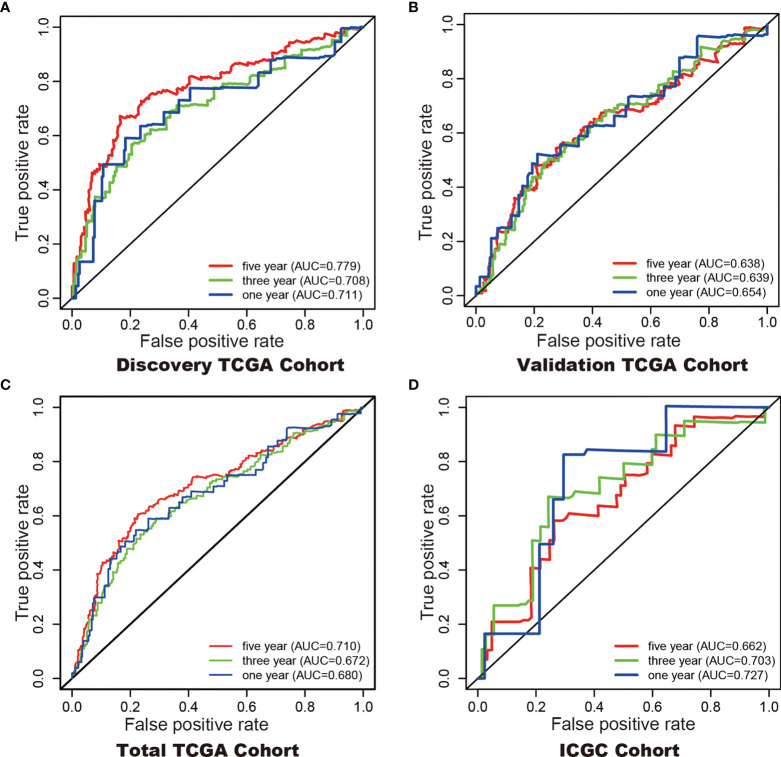
ROC curves with calculated AUCs were established to evaluate the prognostic value of the 6-gene model in 5-, 3-, 1-years in the Discovery TCGA Cohort **(A)**, the Validation TCGA Cohort **(B)**, the Total TCGA Cohort **(C)**, and the ICGC Cohort **(D)**.

**Figure 6:**
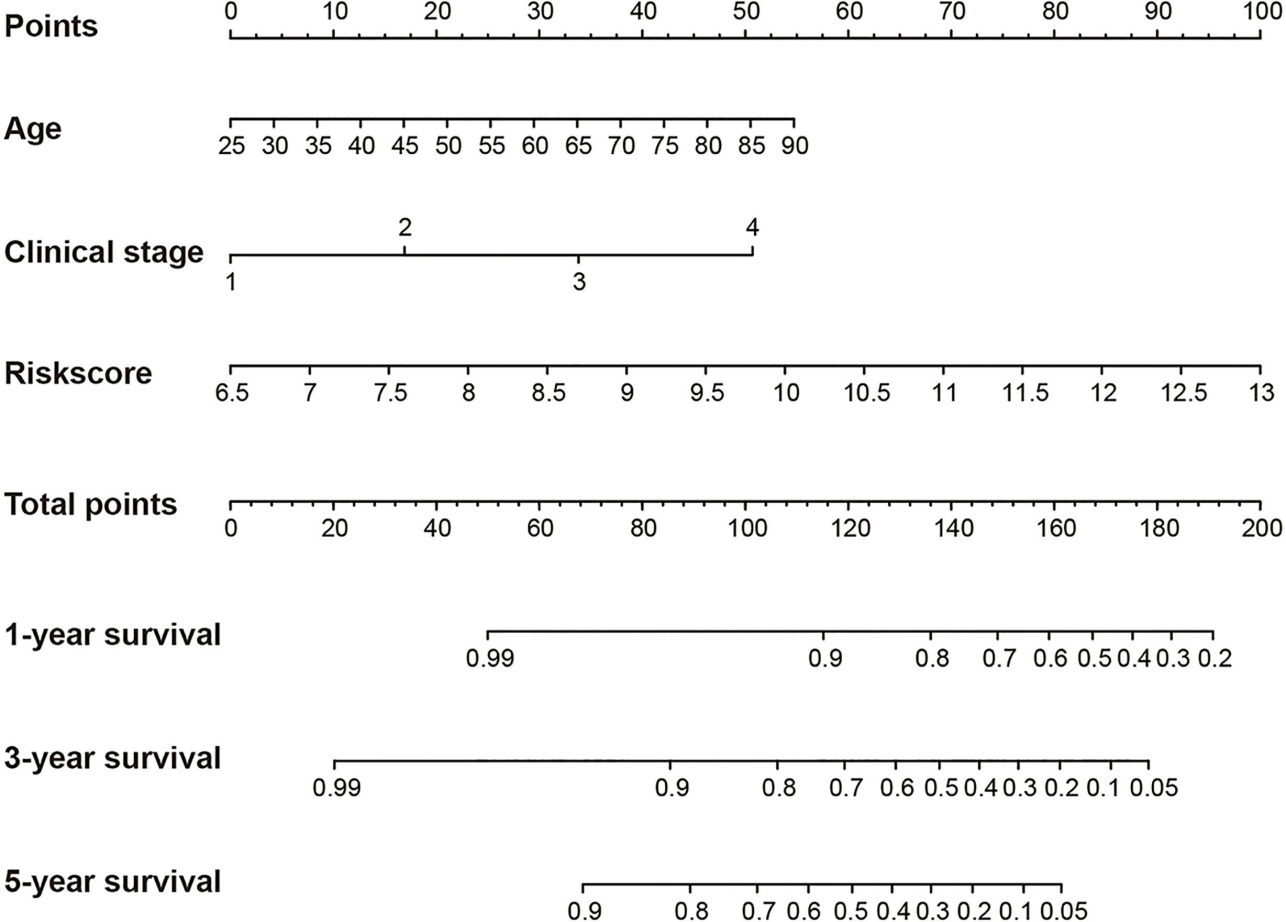
Construction of a nomogram for overall survival prediction in KIRC. The nomogram consists of age, clinical stage and the riskscore based on the six-hypoxia gene model.

In addition, Cox regression analysis remained that riskscore was an independent prognostic factor influencing patients with ccRCC in these four cohorts (**Figure 4**, **Table 1**). The AUC value for the DEHG Prognosis model was 0.711 in the 1-year ROC curve, 0.708 in the 3-year ROC curve, and 0.779 in the 5-year ROC curve of discovery TCGA cohort (**Figure 5A**). The AUC value of the other three validation cohorts is shown in **Figures 5B–D**. The riskscore, patients’ age, and clinical stage could be an independent prognostic factor, respectively, for OS prediction of ccRCC patients in the training set after the univariate and multivariate Cox proportional hazards regression analyses. Then, these independent prognostic factors were integrated together into this nomogram to predict the 1-, 3-, and 5-year OS of ccRCC patients (**Figure 6**).

**Table 1:** Univariate and Multivariate cox regression in the Discovery and Validation cohorts.

Cohorts	Variables	Univariate Cox Regression		Variables	Multivariate Cox Regression	
		HR (95%CI)	p-value		HR (95%CI)	p-value
**Discovery TCGA cohort**	Age	1.028 (1.009–1.047)	**0.003**	Age	1.024 (1.005–1.044)	**0.014**
	Gender	1.025 (0.651–1.612)	0.916			
	Clinical stage	1.885 (1.556–2.284)	**<0.001**	Clinical stage	1.817 (1.269–2.602)	**0.001**
	T stage	1.893 (1.487–2.409)	**<0.001**	T stage	0.823 (0.552–1.227)	0.338
	M stage	1.957 (1.392–2.752)	**<0.001**	M stage	1.258 (0.763–2.075)	0.369
	N stage	0.941 (0.75–1.18)	0.598			
	Riskscore	2.718 (2.056–3.594)	**<0.001**	Riskscore	2.366 (1.73–3.235)	**<0.001**
**Validation TCGA cohort**	Age	1.03 (1.01–1.051)	**0.003**	Age	1.038 (1.016–1.061)	**0.001**
	Gender	1.124 (0.709–1.784)	0.618			
	Clinical stage	1.984 (1.633–2.41)	**<0.001**	Clinical stage	3.072 (1.559–6.053)	**0.001**
	T stage	2.12 (1.657–2.713)	**<0.001**	T stage	0.618 (0.309–1.237)	0.174
	M stage	2.407 (1.724–3.362)	**<0.001**	M stage	0.676 (0.32–1.428)	0.305
	N stage	0.772 (0.622–0.957)	**0.019**	N stage	0.77 (0.619–0.958)	**0.019**
	Riskscore	1.55 (1.21–1.986)	**0.001**	Riskscore	1.515 (1.147–2.001)	**0.003**
**Total TCGA cohort**	Age	1.029 (1.016–1.043)	**<0.001**	Age	1.031 (1.017–1.046)	**<0.001**
	Gender	1.045 (0.758–1.439)	0.789			
	Clinical stage	1.939 (1.693–2.221)	**<0.001**	Clinical stage	2.17 (1.590–2.96)	**<0.001**
	T stage	1.993 (1.683–2.361)	**<0.001**	T stage	0.768 (0.554–1.064)	0.113
	M stage	2.145 (1.696–2.713)	**<0.001**	M stage	1.015 (0.683–1.509)	0.941
	N stage	0.859 (0.735–1.004)	0.057			
	Riskscore	1.975 (1.645–2.37)	**<0.001**	Riskscore	1.777 (1.455–2.17)	**<0.001**
**ICGC cohort**	Age	1.031 (0.993–1.071)	0.109			
	Gender	0.939 (0.456–1.933)	0.863			
	Clinical stage	2.094 (1.515–2.896)	**<0.001**	Clinical stage	2.116 (0.997–4.49)	0.051
	T stage	1.989 (1.402–2.821)	**<0.001**	T stage	0.882 (0.401–1.941)	0.755
	M stage	2.522 (1.394–4.562)	**0.002**	M stage	1.411 (0.54–3.686)	0.482
	N stage	1.162 (0.696–1.938)	0.566			
	Riskscore	1.087 (1.023–1.154)	**0.007**	Riskscore	1.083 (1.018–1.153)	**0.012**

The bold values indicated the correspondence variable was significant difference in Univariates Cox regression or Multivariate Cox regression analysis.

### The Probably Benefit of Patients for Immune Checkpoint Inhibitor (ICI) Therapy in Different Risk Groups

**Figure 7:**
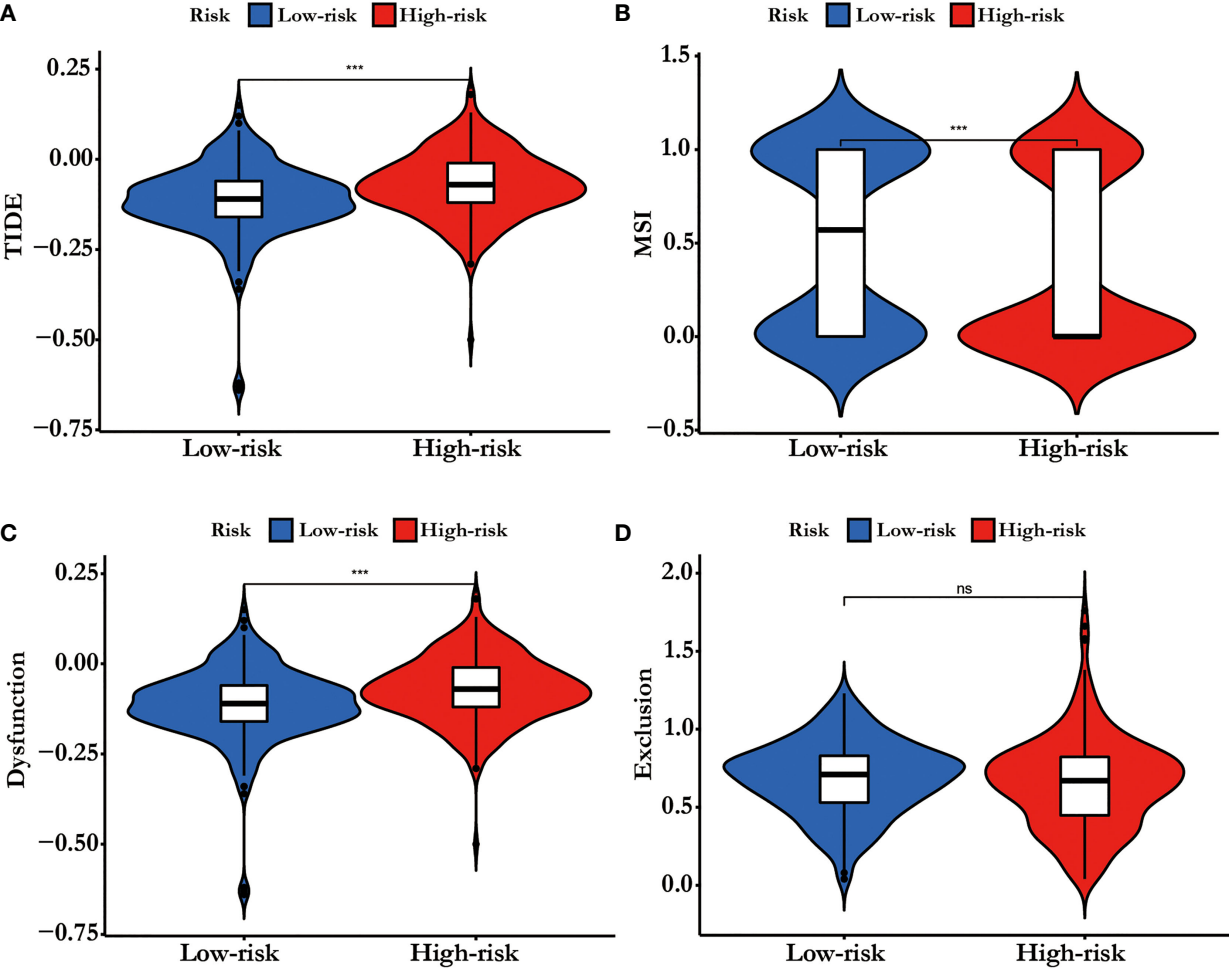
**(A)** TIDE score, **(B)** MSI, **(C)** T cell Dysfunction and **(D)** Exclusion in different risk-groups. The score between the two risk-groups were compared through Wilcoxon test (ns, not significant, ***p < 0.001).

The TIDE-score was used to assess the potential clinical efficacy of immunotherapy in different risk groups. In our results, the high-risk group had a higher TIDE score than the low risk-group (**Figure 7A**). Also, we found that the high risk-group had a lower microsatellite instability (MSI) score, while the high risk-group had a higher T cell dysfunction score, but there was no difference in T cell exclusion between the two groups (**Figures 7B–D**).

## Discussion

To achieve the precision medicine of kidney cancer, accurate diagnosis, individualized therapeutic strategies are needed. Providing the detail prognosis information for patients make the precision medicine more meaningful. Hypoxia pathway plays the central function in ccRCC pathogenesis and development. In our study, we screened the hypoxia-related genes for predicting the prognosis of ccRCC and then constructed a six hypoxia-related gene prognosis model using the LASSO regression method. The model was validated by the validation TCGA cohort, the total TCGA cohort, and the ICGC cohort. A nomogram, based on the clinical features and our model, was built for predicting the overall survival probability of ccRCC patients. In addition, we found the different immune function statuses in the different risk groups.

Recently, prognostic model integrating hypoxia-related gene expression and clinical features emerged (14–16). In our study, the hypoxia-related genes prognosis model was made up of six genes: *GPC3*, *KIF5A*, *PLAUR*, *ANKZF1*, *ETS1*, and *SELENBP1*. In the calculation formula of riskscore, the coefficient of ETS1 was a negative numbers, while the coefficient of the other five genes was a positive number. Therefore, there was a negative relationship between riskscore and ETS1, while there was a positive relationship between riskscore and GPC3, KIF5A, PLAUR, ANKZF1, and SELENBP1. Glypican 3 (GPC3) is an oncofetal proteoglycan anchored on the cell membrane (17). High GPC3 expression was also extensively associated with worse tumor differentiation, later tumor stage, presence of vascular invasion, and hepatitis B virus (HBV) infection (18). This protein is expressed in the liver and the kidney of healthy fetuses but is hardly expressed in adults, except in the placenta. Contrarily, GPC3 is specifically expressed in hepatocellular carcinoma (HCC), ovarian clear cell carcinoma, melanoma, squamous cell carcinoma of the lung, hepatoblastoma, nephroblastoma (Wilms tumor), yolk sac tumor, and some pediatric cancers (19). In addition, GPC3 has a low expression in ccRCC tissues than normal kidney tissues, and it can reduce the proliferation of RCC cell lines (20). Kinesin family member 5A (KIF5A) is a member of the kinesin superfamily which can modulate many cell physiological behavior such as the proliferation of cell cycle (21). KIF5A mutation causes familial Amyotrophic lateral sclerosis (ALS; OMIM: 05400) (22). Tian et al. have proved that KIF5A can regulate the bladder cancer development and progression (21). Plasminogen activator, urokinase receptor (*PLAUR*) encodes the receptor for urokinase plasminogen activator and is a key molecule in regulating of cell-surface plasminogen activation (23). Previous studies have shown that PLAUR was involved in cancer cell migration, invasion and metastasis processing and could predict the prognosis of many cancers, such as glioma and oral squamous cell carcinoma (24–27). Ankyrin repeat and zinc finger peptidyl tRNA hydrolase 1 (ANKZF1) is essential for the oxidative stress and the maintenance of mitochondrial integrity (28). Some bioinformatics analyses have proved that ANKZF1 based genes signature can predict the prognosis of patients with colon cancer, renal cell carcinoma, and prostate cancer (14, 29–33). ETS proto-oncogene 1, transcription factor (ETS1) is a transcription factor belong to the ETS domain family. Ets1 expression is linked to poor survival of some cancers and contributes to the cancer cell invasiveness, EMT, and drug resistance. In addition, as a major MAPK downstream molecule, ETS1 might be a prospective therapeutic target for cancer (34, 35). Moreover, high expression levels of ETS1 were associated with poor survival in RCC tissues (36). Selenium binding protein 1 (SELENBP1) encodes protein and plays a selenium-dependent role in many physiological functions, such as protein degradation, cell differentiation and redox modulation. SELENBP1 is downregulated in many cancer types and its low expression levels are associated with poorer prognosis (37). In RCC, SELENBP1 has been proved as a tumor suppressor gene and low SELENBP1 mRNA expression predicts a worse cancer-specific survival (38).

Although tumor stage, lymph node metastasis and grade are significantly associated with the prognosis of KIRC (39), prognostic model integrating gene expression and clinical features are gained increasing attention along with the development of sequencing technology. Here, we developed a hypoxia-related genes prognostic model and a significant difference in overall survival was observed between high-and low-risk subgroups. The 1- (TCGA 0.68, ICGC 0.72), 3- (TCGA 0.67, ICGC 0.70), and 5- (TCGA 0.71, ICGC 0.66) year AUC showed a good accuracy of ROC curves in the TCGA cohort and the ICGC cohort, respectively. Hypoxia-related genes can be promising prognostic biomarkers for KIRC which gain evidence for hypoxia-related genes targeted therapy.

TIDE score could predict patient response to immunotherapy as it can reflect the potential capacity for the tumor's immune evasion (13).In our results, the high risk-group had a higher TIDE score than the low risk-group, indicating that patients with low riskscore could benefit more from ICI therapy than patients with high riskscore (**Figure 7A**). In addition, high TIDE score predicted a worse outcome, which was accordance with our results and it might interpret the high riskscore group presented a worse prognosis .

In conclusion, a six hypoxia-related gene risk model for independent prediction of the prognosis of KIRC patients was established. The different riskscores might be a biomarker for immunotherapy strategy.

## Data Availability Statement

The datasets presented in this study can be found in online repositories. The names of the repository/repositories and accession number(s) can be found in the article/**Supplementary Material**.

## Ethics Statement

The studies involving human participants were reviewed and approved by the TCGA Research Network: http://cancergenome.nih.gov/. The patients/participants provided their written informed consent to participate in this study. Written informed consent was obtained from the individual(s) for the publication of any potentially identifiable images or data included in this article.

## Author Contributions

X-hN and J-jY conceived and designed the experiment. X-hN, Y-yQ, N-yL, S-cL, and Z-kJ performed the experiments and analyzed the data. Interpretation of the findings were done by X-hN, Y-yQ, and Z-kJ. All authors contributed to the article and approved the submitted version.

## Funding

This work was supported by the Postdoctoral Research Grant in Henan Province (grant numbers 1901004, 1902005), the Henan Science and Technology Research Program (grant number 2018020142) and The Natural Science Foundation of Henan Province (212300410265). The funders had no role in study design, data collection and analysis, decision to publish, or preparation of the manuscript.

## Conflict of Interest

The authors declare that the research was conducted in the absence of any commercial or financial relationships that could be construed as a potential conflict of interest.

## Publisher’s Note

All claims expressed in this article are solely those of the authors and do not necessarily represent those of their affiliated organizations, or those of the publisher, the editors and the reviewers. Any product that may be evaluated in this article, or claim that may be made by its manufacturer, is not guaranteed or endorsed by the publisher.
