# Supplementary material for: Identification of a Hypoxia-Related Gene Model for Predicting the Prognosis and Formulating the Treatment Strategies in Kidney Renal Clear Cell Carcinoma
Source: Front Oncol. 2022 Jan 24;11:806264. doi: 10.3389/fonc.2021.806264 (PMC8818738; doi:10.3389/fonc.2021.806264)
Supplement: Supplementary file 1 [file DataSheet_1.docx]

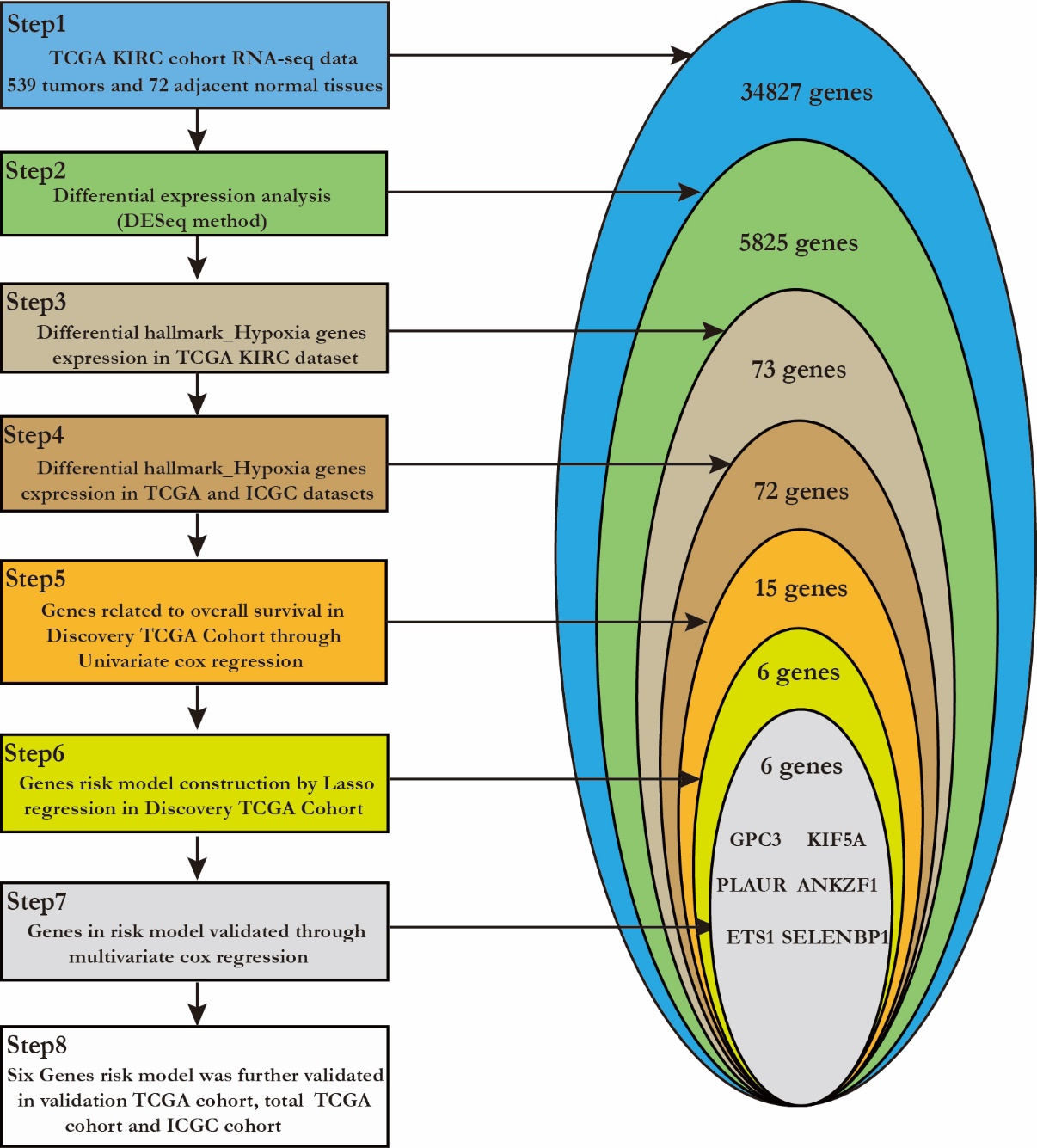


Supplementary Figure 1. The flowchart shows the process/procedure of the establishment and validation of the prognostic model.
