# Supplementary material for: Identification of a Hypoxia-Related Gene Model for Predicting the Prognosis and Formulating the Treatment Strategies in Kidney Renal Clear Cell Carcinoma
Source: Front Oncol. 2022 Jan 24;11:806264. doi: 10.3389/fonc.2021.806264 (PMC8818738; doi:10.3389/fonc.2021.806264)
Supplement: Supplementary file 3 [file DataSheet_3.docx]

| **Variables** | **Discovery TCGA cohort (n=256)** | **Validation TCGA cohort (n=256)** | **Total TCGA cohort (n=512)** | **ICGC cohort (n=91)** |
| --- | --- | --- | --- | --- |
| **Age (years)** | 59(32-88) | 61(26-88) | 60(26-88) | 60(35-83) |
| **Gender** |  |  |  |  |
| **Male** | 155(60.5%） | 181(70.7%） | 336(65.6%） | 52(57.1%） |
| **Female** | 101(39.5%） | 75(39.3%） | 176(34.4%） | 39(42.9%） |
| **Clinical stage** |  |  |  |  |
| **Stage I** | 136(53.1%) | 119(46.5%) | 255(49.8%) | 53(58.2%) |
| **Stage II** | 27(10.5%) | 26(10.2%) | 53(10.4%) | 13(14.3%) |
| **Stage III** | 59(23.0%) | 63(24.6%) | 122(23.8%) | 16(17.6%) |
| **Stage IV** | 34(13.3%) | 48(18.7%) | 82(16.0%) | 9(9.9%) |
| **T stage** |  |  |  |  |
| **T1** | 141(55.1%) | 120(46.9%) | 261(51.0%) | 54(59.3%) |
| **T2** | 31(12.1%) | 34(13.3%) | 65(12.7%) | 13(14.3%) |
| **T3** | 79(30.9%) | 96(37.5%) | 175(34.2%) | 22(24.2%) |
| **T4** | 5(1.9%) | 6(2.3%) | 11(2.1%) | 2(2.2%) |
| **M stage** |  |  |  |  |
| **M0** | 208(81.3%) | 201(78.5%) | 409(79.9%) | 81(89.0%) |
| **M1** | 34(13.3%) | 43(16.8%) | 77(15.0%) | 9(9.9%) |
| **Mx** | 14(5.4%) | 12(4.7%) | 26(5.1%) | 1(1.1%) |
| **N stage** |  |  |  |  |
| **N0** | 125(48.8%) | 108(42.2%) | 233(45.5%) | 79(86.8%) |
| **N1** | 6(2.3%) | 8(3.1%) | 14(2.7%) | 2(2.2%) |
| **Nx** | 125(48.8%) | 140(54.7%) | 265(51.8%) | 10(11.0%) |
| **Survival time(years)** | 3.12(0.01-9.4) | 2.61(0.01-10.0) | 3.04(0.01-10.0) | 4.8(0.01-6.2) |
| **Survival status** |  |  |  |  |
| **Live** | 179(69.9%) | 171(66.8%) | 350(68.4%) | 61(67.0%) |
| **Dead** | 77(30.1%) | 85(33.2%) | 162(31.6%) | 30(33.0%) |
